# Supplementary material for: Conditioned haptic perception for 3D localization of nodules in soft tissue palpation with a variable stiffness probe
Source: PLoS One. 2020 Aug 11;15(8):e0237379. doi: 10.1371/journal.pone.0237379 (PMC7419002; doi:10.1371/journal.pone.0237379)
Supplement: S1 Appendix — This appendix provides the details of the statistical analysis performed to compare the distribution of the force peak prominence for different stiffnesses. (PDF) [file pone.0237379.s001.pdf]

S1 Appendix: Statistical Analysis

1 Introduction

In this document, we present a detailed statistical analysis of the significance of stiffness joint variation on the force peak prominence distribution. The presented statistical analysis tests the null hypothesis that the data from 2 different stiffnesses are coming from the same distribution. Since the distribution for each stiffness is not normally distributed we use the Kruskal-Wallis test, which is particularly suitable for non-parametric distributions. The aim of this study is to study the significance of the probe's stiffness variation on the force peak prominence distributions.

2 Statistical Analysis

2.1 Statistical methods:

- The statistical analysis was performed with the Statistics and Machine Learning Toolbox of MATLAB R2019b.
- The presented statistical analysis has been performed using a Kruskal-Wallis test with the Matlab function kruskalwallis().

2.2 Statistical reporting:

- This study doesn't use pre-processed data.
- The full data set was evaluated; outliers were not removed.
- The threshold for significance (alpha) is 0.05.
- The sample size for each stiffness and each nodule depth is 25.
- For simplicity, the data was stored as a matrix (one per nodule depth) with each column corresponding to one stiffness and the rows correspond to the different trials. Therefore, the Kruskal Wallis tests have been run to compare every column to each other. The null hypothesis is that the data in each column of the matrix comes from the same distribution.

3 Results

Fig 1 summarized the results obtained from the Kruskal Wallis tests. These results show that for the longitudinal sweeps with nodules, the variation of the stiffness of the probe significantly modifies the force peak prominence distribution (p-value$<$ 0.05) in most of the cases. Furthermore, the more different the two stiffnesses are (on Fig 1 the further from the diagonal), the higher the significance of the variation of the force peak prominence distribution is (the lower the p-value is).

However, the results from the Kruskal-Wallis tests for data from the lateral sweeps show that the difference between the force peak prominence's distributions is not statistically significant. These results support our claim that the stiffness variation has a lower significance for the lateral sweeps. Finally, the results of the Kruskal-Wallis of the data from the longitudinal sweep when no nodule is embedded exhibits a lower number of statistically different distributions compared to those with a nodule. This can be interpreted as the fact that the stiffness variation has more impact when there is a nodule to detect, and that in absence of nodule the change of stiffness would not change significantly the force peak prominence distribution.

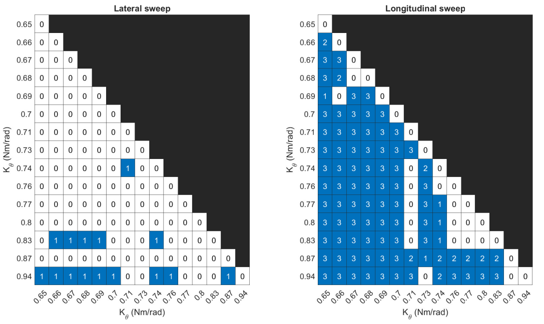

(a) Nodule depth 2 mm

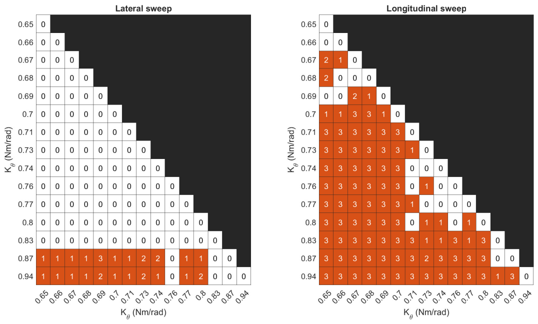

(b) Nodule depth 4 mm

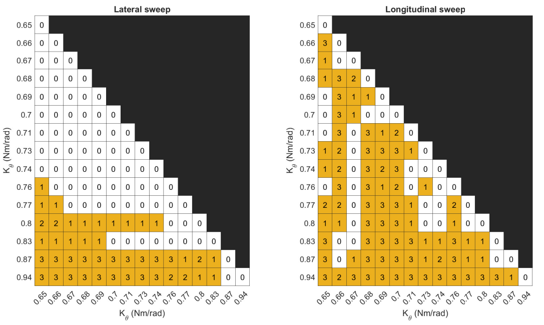

(c) Nodule depth 6 mm

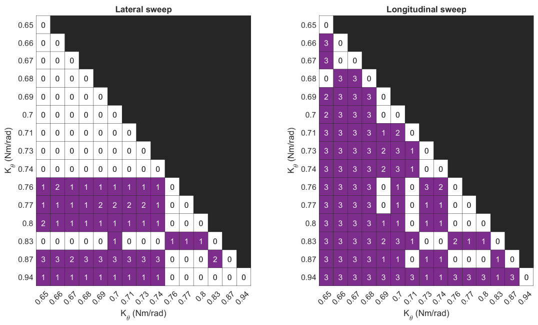

(d) Nodule depth 8 mm

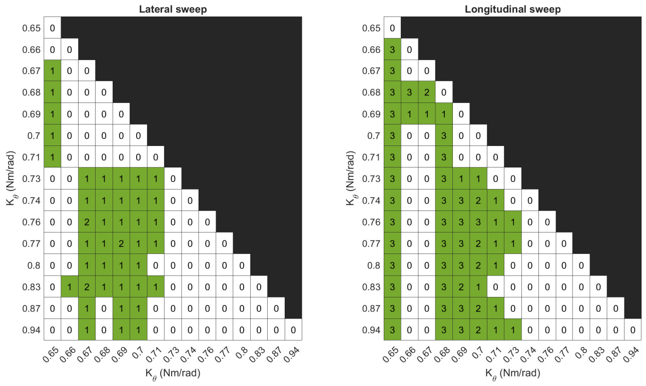

(e) No nodule

Figure 1: Statistical significance results from the Kruskal-Wallis tests to compare the distributions of the force peak prominence across stiffnesses. The colored cells are the one where the difference between the distributions of the force peak prominence of the 2 stiffnesses (x and y axis) are statistically significant (p-value< 0.05). The numbers in the cells correspond to the level of confidence of the Null hypothesis rejection: 0 is for non statistically significant (p-value ≥ 0.05), 1 is for p-value < 0.05, 2 is for p-value < 0.01 and 3 is for p-value < 0.001.
