# Supplementary material for: Conditioned haptic perception for 3D localization of nodules in soft tissue palpation with a variable stiffness probe
Source: PLoS One. 2020 Aug 11;15(8):e0237379. doi: 10.1371/journal.pone.0237379 (PMC7419002; doi:10.1371/journal.pone.0237379)
Supplement: Attachment — Submitted filename: Response to Reviewers.pdf [file pone.0237379.s007.pdf]

Response to Reviewers

Nicolas Herzig, Liang He, Perla Maiolino, Sara-Adela Abad, Thrishantha Nanayakkara,

May 7, 2020

1 Response to the Editor comments

The paper is of interest and addresses the important issue of robotic detection and localization of hard nodules in soft tissues. However, all reviewers agreed that the paper need improvents in terms of rigor of experiments and clarity of presentation. In addition, Reviewer 3 raises important points related to the use of a variable stiffness mechanism for this specific application and some discrepancies between conclusions derived from the experiments versus the ones from FEM.
First, the authors would like to thank the Editorial Board and Reviewers for their time, interest in our paper, and productive comments. All the comments of the reviewers have been addressed in detail in the following document. In particular, we have improved the discussion related to our experimental results, we have also added a statistical analysis to better support our claims and finally, we clarified the presentation of the methods.

2 Response to the Reviewer 1

1. Line 5: Authors reported "This trend is opening up new opportunities for robotic applications in the healthcare field." So, it is implied that the healthcare field with robotics is all about tactile feedback, or all the applications include tactile feedback, which is not true at all. It is true that with the tactile feedback, the robotic implications in healthcare field can be improved significantly - but authors should change their original sentence.

   The authors would like to thank the reviewer for this comment. The authors did not mean that it is the only approach that contributes to healthcare robotics. To avoid confusion, the authors rephrased the sentence as follows:

   In section *Introduction*, paragraph 1 "*This trend is one of the promising advances that can bring new opportunities for robotic applications in the healthcare field.*"

2. Line 37: what do the authors mean by " force and tactile" modalities? Kinesthetic and haptic? Please elaborate or clear these modalities.

   The authors effectively were meaning kinesthetic instead of force. This has been replaced in the highlighted sentence but also in the rest of the paper. In order to clarify this point, the authors have also added these definitions:

   In section *Related work*, paragraph 2 "*The main types of sensors used to do so are the kinesthetic sensors and tactile sensors. In this paper, we refer to kinesthetic sensors, the sensors that aim to give a signal related to the force or the torque applied at a probe joint level. On the other hand, the tactile sensing is referring to fingertip contact sensing using taxel images. Tactile sensing is usually representing the behavior of the mechanoreceptor at the skin level.*"

3. Line 147: The previous sentence cites 4 different studies as the previous studies. Then, the authors start talking about presumably one of them saying "In the previous study ......" without specifying which study they are talking about. It should be clarified.

   As suggested by the reviewer, the authors are now specifying the study that they were talking about:

In section *Related work*, paragraph 9 ”*In the previous study [47], we have shown that the stiffness of the arm and hand joints is modified during the longitudinal sweeping exploration of soft tissues by varying the level of co-contraction of antagonistic muscles.*”

4. The VLM probe explains the design of the probe, which can be supported also visually. The directions and the definitions should be depicted clearly for the reader.

   If the authors understood well the comment, the reviewer is asking for a figure describing clearly the design of the probe and the 2 sweeping directions. The authors have modified Figures 1 and 2. The modified Fig 1 describes the VLM probe design while the modified Fig 2 clearly depicts the 2 sweeping directions. We also revised the definitions in the Materials and methods section to clarify the description of the sweeping directions. The modifications regarding the sweeping directions are detailed in the comment 7. The modifications regarding the design are the following:

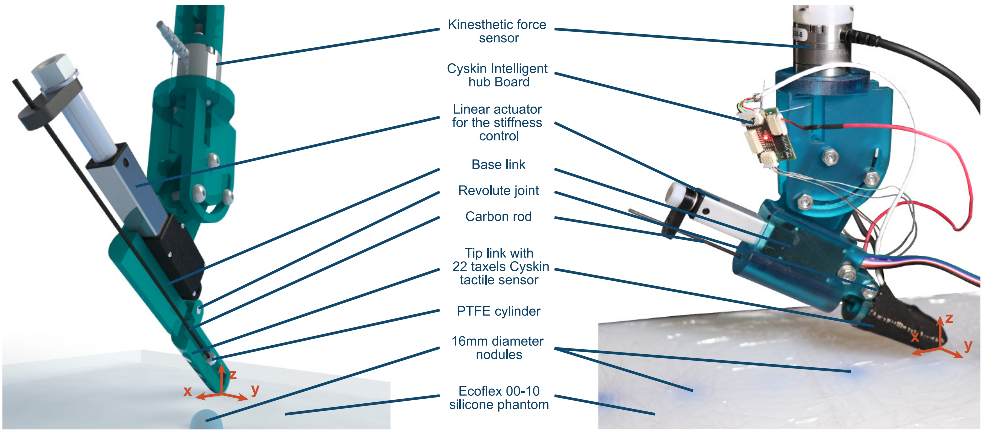

Figure 1: **VLM probe and phantom.** Left: illustration showing the Variable lever mechanism of the VLM probe with a mid-sagittal cut of the tip link and base link. Right: VLM probe and phantom with nodules with different depths

In section *Variable stiffness palpation: the VLM probe*, paragraph 2 ”*Fig 1 shows the design of the VLM probe. The VLM probe is based on a revolute variable stiffness joint composed of 2 rigid links (the base link and the tip link) connected with a revolute joint in parallel with a deformable carbon rod. This carbon rod acts as a variable spring that allows the stiffness of the joint to be controlled thanks to an* Actuonix L12-30-50-6-I *linear actuator. This actuator slides the carbon rod through the base link and the tip link changing the length of the carbon rod that can be bent (active length). As one can see, the hole in the base link has been designed such as that the carbon rod can slide axially but is constrained radially to prevent bending of the rod in the base link. On the other hand, the hole in the tip link is large enough to allow the carbon rod to bend in. A PTFE cylinder is used to transmit the radial forces between the tip link to the carbon rod. This PTFE cylinder has been designed to slide easily axially when the actuator is translating the carbon rod. Adjusting the active length of the carbon rod changes, by cantilever effect, the amount of force required to bend the rod and by consequence the angular stiffness of the probe.*”

In section *Variable stiffness palpation: the VLM probe*, paragraph 3 ”*In order to describe the movement of the probe, we need to define a frame. First, we define the axis* **z** *as the direction of the normal to the phantom surface. We then define the* **x** *axis as the intersection between the tangent surface of the phantom and the mid-sagittal plane of the probe. Finally, the* **y** *is defined in order to obtain a direct orthonormal frame (***x***,***y***,***z***). In the rest of the paper, this reference frame will be used to describe the directions of forces or displacement.*”

5. The pdf version I received had the actual Figure and the figure captions separated. I am not sure if this was a draft problem, or the final manuscript will be like this. If it’s the later, numbering the sections make no sense, because the reader cannot follow. The authors should find a way to label the system parts on the images directly. Also, using the arrows with color code based on the motion direction might be impossible for the reader to capture, if they are reading from a black/white copy. Such an identification should be handled differently.

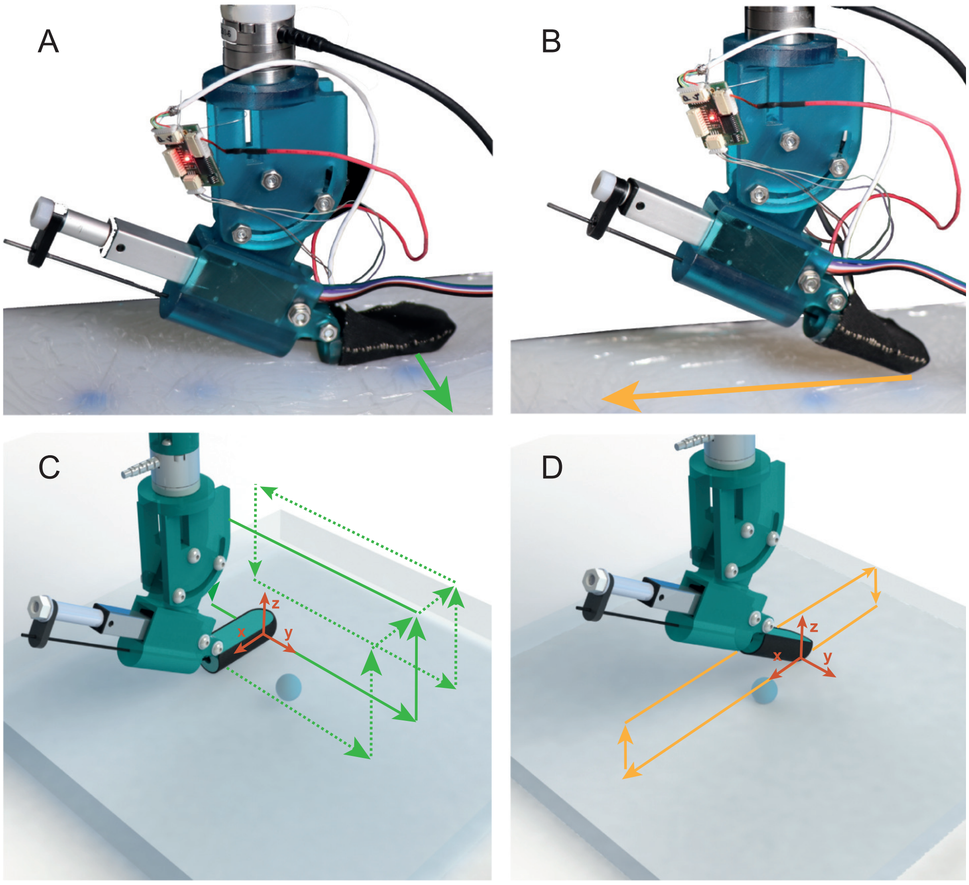

Figure 2: **Sweeping directions.** (A) Picture taken during a lateral sweep. (B) Picture taken during a longitudinal sweep. (C) Probe trajectory during lateral sweeps with different shifts. (D) Probe trajectory during longitudinal sweeps

The figures were separated from the captions as explicitly defined in the PLOS submission guideline, the final manuscript once edited should integrate the figures in the text. However, in order to simplify the review process, the figures with section numbers have been edited. Also, the caption referring to colors have been changed to help the reading from grayscale copies.

6. Why exactly Figure 1 (a) and (b) have different coordinate systems? I understand in both conditions, the sweeping action takes place in the y direction but what does that mean? What is the advantage of such rotation for the designer?

   The difference in the coordinate system was a mistake. Figures 1 and 2 have been modified to avoid confusion with the reference frame. More details about the motivation of having the two sweeping directions are given in the next answer.

7. The motivation of having two conditions in Figure 1(a) and (b) are not clear. So, the direction of sweeping are different, but they are still tangential to the surface. What is the hypothesis or the expected outcome here? To have different ”depth estimation”, changing the direction of tangential sweep might be not enough.

   The authors thank the reviewer for this comment. The aim of having two different sweeping strategies is to reproduce the exploration behavior of human participants observed during the previous study [44]. Indeed we observed that different strategies could be applied to localize the nodule and to estimate the depth. In particular, this study showed that the force applied during the palpation varies according to the aim of the exploration. Based on these observations and results we found interesting to compare the two type of sweeping strategies, one local with a light force applied to the phantom and one more global (with the whole palmar region of the probe’s finger). The hypothesis behind the interest of the sweeping direction is that one direction is more suitable for nodule localization in the tangential plane where the

other one gives better results to estimate the nodule depth. This hypothesis has been verified through the study. The authors agree with the fact that changing the direction of the tangential sweep is not the only impacting factor for depth estimation. The presented paper discusses the interest of stiffness variation as well. To clarify the information regarding the two sweeping directions, the following modification has been added to the paper:

In section *Sweeping directions*, paragraph 2 "*The aim of the two sweeping directions is to reproduce some human participants' palpation strategies that we observed during our previous study [44]. We have shown in this study that the palpation behavior of the participants is adapted to localize the nodule or to estimate the depth. From these observations and results, we found interesting to compare two types of sweeping strategies, one local with a light force applied to the phantom using the tip of the probe and one more global using the whole palmar region of the probe with the tactile sensor.*"

8. Also, given Figures have different orientations of component (6), resulting different contact areas with the surface. Is it intentional? If so, how is it related to the sweep direction? If not, why is it different?

   The authors thank the reviewer for this comment. On figure 1, the orientation of the phantom and nodule (previously labelled (6)) was, effectively, different between the subfigure (a) and (b). The proposed probing strategy aims to detect the 3D location of the nodules independently from the orientation of the phantom. This is also the reason why the phantom was also examined in different orientations during the evaluation of the algorithm. In this regard, the two sweeping directions have been tested for different orientations of the phantom. The modification of Figures 1 and 2 (presented in our answer to comment 4 of the reviewer 1) should suppress the confusion. To clarify that the strategy aims to be independent of the initial phantom orientation, the following sentence has been added in the paper:

   In section *Evaluation of the algorithm*, paragraph 1 "*Finally, the proposed palpation strategy aims to localize the nodule independently from the phantom orientation, so the algorithm has been tested for several orientations of the phantom.*"

9. Figure 2 is impossible to be understood - possibly because the coordinate system for both conditions are different. Still, this Figure must me improved!

   Fig 2 has been modified to improve clarity. The latter is presented in our answer for your comment 4.

10. The probe position seems to be changing between trials in the lateral sweep, but not in the longitudinal sweep. Why?

    As explained in previous answers, the two sweeping directions have different objectives. The interest of the lateral sweep is to detect the position of the nodule in the tangential plane. To localize the nodule in the (**x**,**y**) plane, the probe utilizes both the kinesthetic sensor and the tactile sensor. Then, once a nodule is localized, the longitudinal sweeping is used to improve the depth estimation thanks to the kinesthetic feedback. In this regard, it is interesting to change the position for the lateral sweep (nodule position detection) but not for the longitudinal sweep (nodule depth estimation). The following paragraph has been rephrased to improve the clarity of the paper.

    In section *Sweeping directions*, paragraph 6 "*This cycle is repeated 5 times, and after the fifth time, the VLM probe is shifted by 5mm along* **x** *axis to a new initial position. As the lateral sweeps are performed to localize nodule on a wide area, the aim of this shift is to observe the behavior of the probe when the latter is sweeping over a nodule at different distances. The next cycle is also repeated 5 times before applying a new shift. In total, 4 shifts are applied, the distance between the initial and last trajectories is then 20mm.*"

11. 15 different stiffness values have been chosen for the experiment and these values seem random. It seems like these values are changing incrementally, but not linearly (there are some missing values) but it is curious how they are chosen! Is there a reason why all the values between 0.65 and 0.71 was tried, but 0.72 is ignored? Why is the differences between the last 4 values are much bigger than the first 4?

    The nonlinearity in the stiffness is coming from the VLM probe behavior. Indeed in a previous study [22], the VLM probe stiffness has been modeled and characterized for several active lengths of the carbon rod. Instead of using constant steps in stiffness, the authors have chosen linear steps of active

length of the carbon rod for three reasons: 1) we are using the same active carbon rod length as the one we characterized in our previous study, 2) it is practically easier to control accurately the position of the actuator thanks to its sensor (closed-loop control) 3) It is simpler to implement carbon rod displacement in the FEM simulation. To clarify this information, the following paragraph has been added.

In section *Sweeping directions*, paragraph 9 "*One can notice that the steps of the stiffness tested in this paper is not linear. This comes from the fact that for simplicity, we have chosen linear steps of* 2*mm in the active length of the carbon rod. This choice allows us to take advantage of the probe characterization performed in our previous study [22] and makes the stiffness control easier by relying on the closed-loop position control of the linear actuator. It also simplifies the implementation of carbon rod displacement in the FE simulation. However, since the relation between the stiffness and the active length of the carbon rod is nonlinear, it results in nonlinear steps of stiffness.*"

12. FE model is only used for the longitudinal sweep but not for the lateral. Why?

The reasons why the FE model for the lateral sweeps is not proposed in the paper are multiple. First, the aim of our FE model is to provide a further study on the impact of the joint stiffness variation during palpation exploration. However, the experimental results show that the stiffness variation for the lateral sweeps is less significant than for the longitudinal sweeps. As a consequence, there was less interest to further carry the FE simulation for the lateral sweep. Second, to model the lateral sweeps, the FE simulation needs to be modified from a 2D model to a 3D model. This implies an exponential increase in computation cost (27 hours were already required to compute the simulation in 2D). Also, to follow the experimental protocol, the simulation should be performed for the five different shifts, which also increase the computational cost of the study. Finally, in the proposed algorithm, the lateral sweep is generally used only once. According to the author, the complexity is not worth the information that the lateral sweep FEM simulation would bring to this paper. To clarify, the following modifications have been added to the paper:

In section *Finite Element Simulation*, paragraph 1 "*The aim of our Finite Element (FE) model is to provide a further study on the impact of the joint stiffness variation during palpation exploration. The experimental results show that the variation of stiffness is more significant for the longitudinal sweeps than for the lateral sweep. As a consequence, we focused our FE simulation on longitudinal sweeps.*"

In section *Discussion*, paragraph 2 "*Also, simulating the lateral sweeps would require developing a 3D FE model and repeating the simulation for several shifts (position along* $\mathbf{x}$*). These modifications would increase the computational cost of the simulation significantly.*"

13. Line 588 : Authors say "This study highlights the role of compliance of a soft robot not only as a design parameter for safety, but also as a control parameter for improved haptic perception " but in the paper, what we see is the comparison between different sweep directions. If these two things are connected, it means authors didn't do a very good job explaining how the sweep direction is related to the probe compliance. It would be also nice to mention this relationship in the discussion and/or conclusion section.

The paper presents the analysis of the impact of the probe stiffness (or compliance) variation on the haptic (kinesthetic and tactile) detection of nodules in soft tissues for two sweeping directions. The authors would like to apologize if the confusion comes from the use of the word compliance. Indeed, compliance is defined as the inverse of the stiffness. The authors then used from time to time compliance instead of stiffness to avoid repetition. To avoid confusion, the authors have added the following modification in the paper:

In section *Introduction*, paragraph 3 "*Since the compliance is defined as the inverse of the stiffness, we mean by compliant system a physical system with low stiffness. By opposition, a stiff system is a system with low compliance. We will deliberately use the two words compliance and stiffness inconsistently in this paper since some ideas are more intuitive when expressed using the stiffness, while some others are more intuitive with the compliance.*"

14. Citation numbers cannot be used as a subject of a sentence ([35] proposed . . . . . .)

As suggested, this has been corrected in the revised manuscript.

In section *Related work*, paragraph 7 ”*Using point by point strategy, Hoshi* et al. *[36] proposed an algorithm to optimize the stiffness estimation of the palpated tissues by coupling the force measurement with a predictive model based on the Finite Element Method*”

3 Response to the Reviewer 2

This paper presents a control algorithm for a variable lever mechanism probe to detect and localize embedded nodules in soft tissues. Using this algorithm, the 3D position of the nodule can be estimated. In general, this paper is well-written. There are some questions and possible improves below.

1. What is the material used to make the simulated nodules? What types of soft tissues and nodules do you simulate?

   The components used to simulate the nodule are acrylic spheres of 16mm diameter. The size of the nodule represents the size of a tumor type T1 ($<$ 2cm) for breast or liver cancer. This is, according to the TNM classification, the earliest stage where the nodules can be detected by palpation [49]. With the platinum-catalyzed silicone (Ecoflex 00-10), the authors aim to simulate general human soft tissues, for instance, abdominal organs such as liver or human breast. Indeed, this type of silicone is widely used to mimic the mechanical properties of human tissues during palpation [22] or needle insertion [48]. This information has been added in the paper

   In section *Soft tissue phantom with nodules*, paragraph 2 ”*These materials have been widely used to simulate human soft tissues mechanical properties. In particular, Ecoflex 0010 has been used in biomedical simulators to practice abdominal palpation [11] or needle insertion [48]. The size of the nodule represents the size of a tumor of type T1 (<2cm) for the breast or liver cancers. This is, according to the TNM classification, the earliest stage where the nodules can be detected by palpation [49].*”

2. How do you detect when the contact between the probe and the phantom starts? How does the accuracy of detection of the moments of the contact between the probe and the phantom affect the stiffness estimation?

   The authors thank the reviewer for this question. The contact between the probe and the phantom is detected every time the probe changes the palpation area thanks to its kinesthetic sensor. As described in the paper, the method is based on an indentation (without sweeping) and the detection of force variation. As shown in Fig 4, from the force sensor readings, it is simple to detect when the probe touches the phantom. Since each actuator has its own position sensors, the position of the probe during the contact can be found. The accuracy of the contact point detection is important; related studies have shown that a variation of indentation can impact the stiffness estimation [47, 25]. The described detection routine and the use of the force peak prominence (which takes into account the force measured around the peak) aims to improve the robustness against an indentation error. More discussion on this point has been added in the paper.

   In section *Variable stiffness palpation: the VLM probe*, paragraph 6 ”*To autonomously detect the* 0*mm indentation position, the method is based on an indentation (without sweeping) and the detection of variation in the kinesthetic sensor measurement. This detection strategy aims to improve the robustness of the nodule detection by improving the accuracy of the indentation measurement. This is particularly important since related studies have shown that a variation of indentation can impact the nodule depth estimation[47, 25].*”

4 Response to the Reviewer 3

This paper addresses the problem of detecting hard nodules in soft tissue via robotic palpation, as well as assessing their depth. The central ideas are one, that the stiffness of the probe should matter; and two, that the proper stiffness can be found via a Bayesian search technique. The paper is clearly written and technically sound; however, I see very little evidence that supports the central ideas.

1. First, a few more details: the variable stiffness probe is mounted on a force sensor and equipped with a tactile sensor. Although Fig 7 shows that the latter provides some useful information, as far as I can tell, it is not used to support any of the paper's main points. Therefore, I see the tactile sensor as a bit of a distraction. I would recommend removing it altogether. In any event, the force sensor appears to give a clear indication of nodule location as well as depth. There is no doubt that the robotic probe succeeds!

   The tactile sensor plays an important role in the nodule localization in the (**x**,**y**) plane. Indeed as explained in the algorithm, the tactile sensor is used to locate the x_n position of the nodule. The detection of x_n after a lateral sweep is not possible from the force sensor only. Without the x position computed after the lateral sweep, the longitudinal sweeping distance would have to be increased by two times the length of the tip of the probe. Increasing this distance would degrade the performances of the proposed method, in terms of time (longer region to probe several times implies a longer time to find the nodule). In this regard, the authors decided to keep the tactile sensor in the paper but added more discussion about it.

   In section *Discussion*, paragraph 5 "*In the proposed algorithm, the tactile sensor is currently used to help to find the location of the nodule. Removing the sensor would be possible, but it would require to increase the longitudinal sweeping distance by two times the length of the probe's tip link. This would then increase the exploration time and also the tissue region area that is probed. These two factors are not really suitable in the case of patient palpation.*"

2. My concern, however, relates to the importance of probe stiffness. Figures 6, 9 and 11 illustrate the dependence of the "force peak prominence" on probe stiffness under lateral and longitudinal swiping, in simulation and experiment. With the exception of Fig 11, we see very little dependence on probe stiffness. Even with Fig 11, it would appear to suffice to pick a good stiffness (lower values appear better) and to fix it. The added value of varying the stiffness is by no means apparent.

   The authors thank the reviewer for this comment. To provide a generic method that a user could reproduce for any controllable stiffness probe, we present a detailed statistical analysis of the significance of joint stiffness variation on the force peak prominence distribution. This statistical analysis supports the authors' claim that the stiffness plays a significant role in the force peak prominence distribution during longitudinal sweeps and by consequence on the nodule depth estimation. Also, the case where a low fix stiffness ($K_\theta = 0.68$Nm/rad) is maintained across trials have been tested and added to the Fig 15 to highlight the limitation of such a strategy in term of estimation confidence.
   The presented statistical analysis tests the null hypothesis that the data from 2 different stiffnesses are coming from the same distribution. Since the distribution for each stiffness is not normally distributed, we use the Kruskal-Wallis test, which is particularly suitable for non-parametric distributions. The results are summarized in the supplemental Appendix S1. These results of cross Kruskal-Wallis tests show that, for the longitudinal sweeps with nodules, the force peak prominence is statistically different (p-value< 0.05). In addition, the more different the two stiffnesses are, the higher the significance of the difference between their force peak prominence distribution is. Furthermore, the results from the Kruskal-Wallis tests for data from the lateral sweeps show that the difference between the force peak prominence's distributions is not statistically significant. These results support our claim that the stiffness variation has a lower significance for the lateral sweeps. Finally, the results of the Kruskal-Wallis tests for the longitudinal sweeps data when no nodule is embedded exhibits a lower number of statistically different distributions than the ones from the data with a nodule. This can be interpreted as the fact that the interaction between the nodule and stiffness impacts the force peak prominence significantly. In order to make clearer that the stiffness variation is more significant for the longitudinal sweeps than for the lateral sweeps, the authors have added the following paragraphs:

   In section *Conclusion*, paragraph 1 "*For the lateral sweep, the impact of the joint's stiffness variation on the force peak prominence distribution is not as significant as for the longitudinal sweeps. This results in the fact that the haptic information gain is not sufficient to distinguish the depth of a nodule. However, the stiffness can be chosen to facilitate the detection of a nodule. In contrast, for the longitudinal sweeps, the haptic information gain from one depth to another is significant and helps to determine which stiffness is suitable for the depth estimation.*"

   In section *Lateral sweep*, paragraph 10 "*To further support the interpretation of Fig 6, we detail, in the*

supplemental Appendix S1, a comparison of the distributions obtained for each stiffness using statistical analysis."

In section *Experimental results*, paragraph 2 "*Similarly to the lateral sweeps, the significance of the probe's stiffness variation on the force peak prominence distribution is further studied, using statistical analysis, in the supplemental Appendix S1.*"

To highlight the interest of the proposed algorithm compared to a palpation strategy with constant stiffness. Two examples of Bayesian nodule depth estimation with a constant stiffness (chosen low as recommended by the reviewer) have been added to the figure 15. These 2 examples clearly show that the information gain (KL divergence) drops quickly, and the final confidence level of the depth estimation stays low but with good accuracy. The discussion added to compare the scenario with a constant stiffness with the proposed algorithm follows:

In section *Evaluation of the algorithm*, paragraph 8 "*In addition, to complement the investigation on the effect of the probe's stiffness variation in the Bayesian nodule depth estimation,* 2 *trials have been run without the stiffness modulation strategy. During these trials performed for* 4 *and* 6*mm nodule depths, the stiffness is therefore maintained constant at the stiffness* $K_\theta = 0.68$*Nm/rad (the same as the one used for lateral sweeps). The results for these trials are shown in Fig 15B. One can see that the accuracy stays similar to the case where the stiffness is updated from previous knowledge but the confidence level is significantly lower. It can also be observed in Fig 15C that the KL divergence quickly converges to* 0*, which means that the repetition of the sweeps with the same fixed stiffness did not bring much information on the nodule depth. These results confirm that the proposed strategy using stiffness variation helps in conditioning the force peak prominence likelihood and improves the nodule depth estimation.*"

3. This brings us to the Bayesian search, in which stiffness was updated trial-over-trial in a Bayesian fashion using likelihood functions at different stiffness values with prominence and nodule depth as variables. Two sets of likelihood functions, one obtained experimentally and one obtained from FEM. In both cases, the Bayesian technique clearly shows the best stiffness varying trial-over-trial. That variation, however, is not good evidence that an optimal stiffness is being obtained. To the contrary, it is notable that the best stiffness versus module depth behaves completely differently in Fig 14 (experimental likelihood) versus Fig 15 (FEM). The former tends toward a softer probe for deeper nodules and the latter tends toward a stiffer probe for deeper nodules. It is deeply concerning that the answers are just the opposite of one another. However, when looking at Fig 13, it appears that the likelihood functions simply don't vary much with stiffness. I suspect that the results are just noise.

The authors thank the reviewer for this comment. This paper shows how a set of favorable likelihood functions can be used for fast convergence of the posterior to a higher information gain. The paper shows that this likelihood function conditioning can be done just by changing the stiffness.
The authors did not claim that a global optimal stiffness exists. Indeed, the claim of the author is even the opposite saying that the stiffness needs to be adapted according to the current knowledge during the palpation exploration. The proposed method then presents a strategy that tunes the stiffness in order to increase the probability of getting new information based on prior knowledge (likelihood functions). Comparing the gradient of the selected stiffness between 2 different trials does not really make sense since it is dependent on the past measurement of a random variable (the force peak prominence). Due to the stochastic behavior of this variable, even for the same stiffness and same nodule, the information obtained is different (which is the purpose of the paper). By consequence, it is not surprising that the stiffness gradient followed during the trials with the likelihood functions obtained from the FEM (Fig 15) and the likelihood functions obtained from experimental data (Fig 14) are different. This comes from the fact that the prior knowledge is different and so is the information remained to be obtained. This can also be supported by another study [14] where the variation of the human's joints stiffness during palpation tasks has been studied and clearly showed that this joint stiffness follows a random walk. This confirms that during palpation exploration tasks even humans do not follow a particular gradient of joint stiffness variation.
Even if in Fig 13, the variation of the likelihood function for different stiffness is not visually obvious, the added statistical analysis clearly shows that the variation of the force peak prominence distribution due to a stiffness variation is statistically significant for the longitudinal sweeps. The discussions obtained

by addressing the reviewer's comments have been used to strengthen the discussions in the paper. The modifications done are the following:

In section *Discussion*, paragraph 6 "*Finally, one may notice that the stiffness variations (for the same nodule depth) during the trials with likelihoods functions computed from experimental do not necessary follow the same gradient as the variations during the trials with likelihoods function computed from the FE simulations. More generally, even across trials repeated for the same scenario, we observed different stiffness variations. This comes from the fact that the stiffness is adapted according to the current knowledge during the palpation exploration. The proposed method then presents a strategy that tunes the stiffness in order to increase the probability of getting new information based on prior knowledge (likelihood functions). Due to the stochastic behavior of the force peak prominence, even for the same stiffness and same nodule, the information obtained during a sweep is different from one sweep to another. As a consequence, for two trials on the same nodule, if the information collected so far is different, the stiffness selected to maximize the information gain of the next sweep will be different. Finally, this can also be supported by another study [14] where the variation of the human's joints stiffness during palpation tasks has been studied and clearly showed that this joint stiffness follows a random walk. In other words, during palpation exploration tasks, even humans do not follow a predictable gradient of joint's stiffness variation.*"

In section *Experimental results*, paragraph 5 "*In particular, the bests of the tested stiffnesses to detect the presence of a nodule in the stiffness range of the VLM probe are respectively* $K_\theta = 0.68$*Nm/rad and* $K_\theta = 0.76$*Nm/rad for the lateral sweep and for the longitudinal sweep.*"to avoid the use of "optimal".

4. Minor point: could the oscillations seen in Figs 8 and 10 be, in part, due to probe dynamics? Perhaps stick-slip excites those dynamics.

   These oscillations come from both the dynamics of the probe and the dynamics of the phantom (both connected in series). Indeed, if they were coming exclusively from the probe dynamics, the oscillations would vary with the stiffness variation but not with the nodule depth. Also, one can see that for the same stiffness, the amplitude of the oscillations is varying with the nodule depth (also visible on simulation from Fig 8); so, both dynamics are important. The authors strongly agree on the fact that the stick-slip of the probe excites the probe and phantom dynamics. For improving clarity, more discussion about these oscillations has been added in the paper.

   In section *Simulation results*, paragraph 3 "*Furthermore, one can notice that the amplitude of these oscillations varies with the nodule depth, which means that these oscillations are not only dependent on the probe's internal dynamics but also on the ones from the phantom.*"

S1 Appendix: Statistical Analysis

1 Introduction

In this document, we present a detailed statistical analysis of the significance of stiffness joint variation on the force peak prominence distribution. The presented statistical analysis tests the null hypothesis that the data from 2 different stiffnesses are coming from the same distribution. Since the distribution for each stiffness is not normally distributed we use the Kruskal-Wallis test, which is particularly suitable for non-parametric distributions. The aim of this study is to study the significance of the probe's stiffness variation on the force peak prominence distributions.

2 Statistical Analysis

2.1 Statistical methods:

- The statistical analysis was performed with the Statistics and Machine Learning Toolbox of MATLAB R2019b.
- The presented statistical analysis has been performed using a Kruskal-Wallis test with the Matlab function kruskalwallis().

2.2 Statistical reporting:

- This study doesn't use pre-processed data.
- The full data set was evaluated; outliers were not removed.
- The threshold for significance (alpha) is 0.05.
- The sample size for each stiffness and each nodule depth is 25.
- For simplicity, the data was stored as a matrix (one per nodule depth) with each column corresponding to one stiffness and the rows correspond to the different trials. Therefore, the Kruskal Wallis tests have been run to compare every column to each other. The null hypothesis is that the data in each column of the matrix comes from the same distribution.

3 Results

Fig 1 summarized the results obtained from the Kruskal Wallis tests. These results show that for the longitudinal sweeps with nodules, the variation of the stiffness of the probe significantly modifies the force peak prominence distribution (p-value$<$ 0.05) in most of the cases. Furthermore, the more different the two stiffnesses are (on Fig 1 the further from the diagonal), the higher the significance of the variation of the force peak prominence distribution is (the lower the p-value is).

However, the results from the Kruskal-Wallis tests for data from the lateral sweeps show that the difference between the force peak prominence's distributions is not statistically significant. These results support our claim that the stiffness variation has a lower significance for the lateral sweeps. Finally, the results of the Kruskal-Wallis of the data from the longitudinal sweep when no nodule is embedded exhibits a lower number of statistically different distributions compared to those with a nodule. This can be interpreted as the fact that the stiffness variation has more impact when there is a nodule to detect, and that in absence of nodule the change of stiffness would not change significantly the force peak prominence distribution.

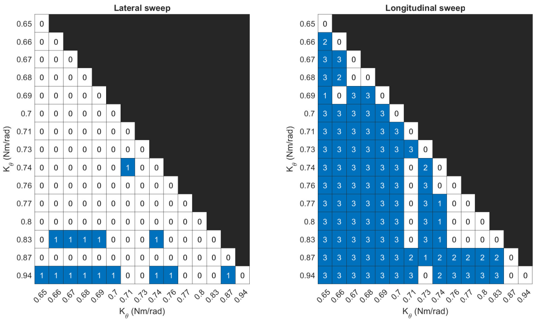

(a) Nodule depth 2 mm

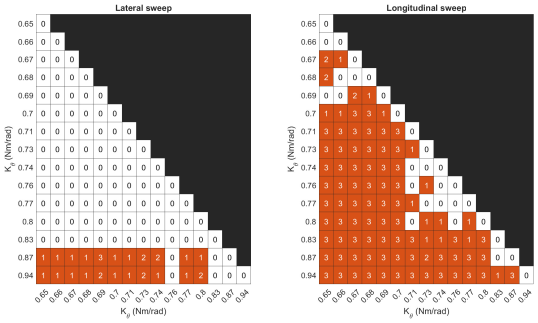

(b) Nodule depth 4 mm

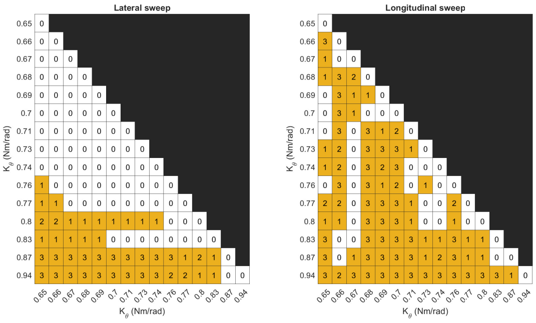

(c) Nodule depth 6 mm

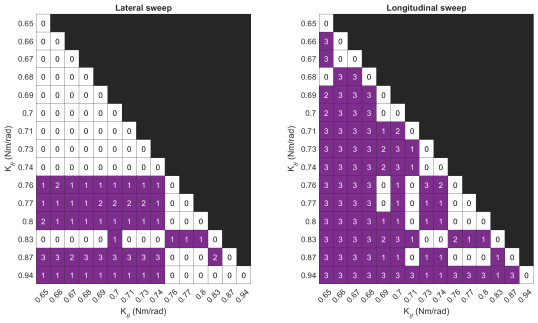

(d) Nodule depth 8 mm

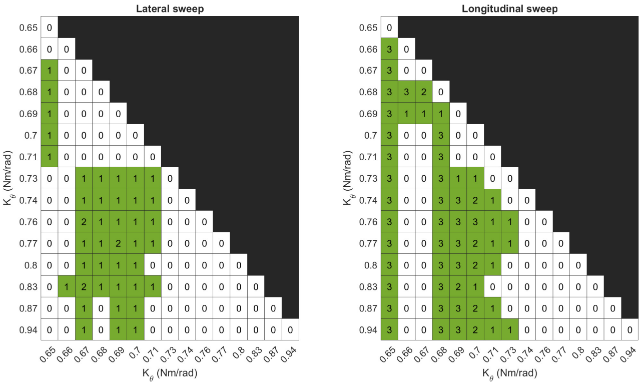

(e) No nodule

Figure 1: Statistical significance results from the Kruskal-Wallis tests to compare the distributions of the force peak prominence across stiffnesses. The colored cells are the one where the difference between the distributions of the force peak prominence of the 2 stiffnesses (x and y axis) are statistically significant (p-value< 0.05). The numbers in the cells correspond to the level of confidence of the Null hypothesis rejection: 0 is for non statistically significant (p-value ≥ 0.05), 1 is for p-value < 0.05, 2 is for p-value < 0.01 and 3 is for p-value < 0.001.
