# Supplementary material for: Conditioned haptic perception for 3D localization of nodules in soft tissue palpation with a variable stiffness probe
Source: PLoS One. 2020 Aug 11;15(8):e0237379. doi: 10.1371/journal.pone.0237379 (PMC7419002; doi:10.1371/journal.pone.0237379)
Supplement: Attachment — Submitted filename: Response to reviewers.pdf [file pone.0237379.s008.pdf]

Response to Reviewers

Nicolas Herzig, Liang He, Perla Maiolino, Sara-Adela Abad, Thrishantha Nanayakkara,

June 29, 2020

1 Response to the Editor comments

Thank you for submitting your manuscript to PLOS ONE. After careful consideration, we feel that it has merit but does not fully meet PLOS ONE's publication criteria as it currently stands. Therefore, we invite you to submit a revised version of the manuscript that addresses the points raised during the review process. The authors should still address the comments from the reviewer in particular on: (1) how the experimental results can be generalized to non-homogeneous curved objects, (2) the validation of the accuracy of the proposed methodology, and (3) the modeling approach.
First, the authors would like to thank the Editorial Board and Reviewers for their time, interest in our paper, and productive comments again. All the points raised during the review process have been addressed in detail in the following document. In particular, we discussed the generalization of the proposed methodology, we validated the accuracy with the root mean square error, and we further clarified the model approach.

2 Response to the Reviewer 3

Thank you for the thorough response to my critique and for adding the constant stiffness case as a comparison. I also stand corrected on the behavior of the stiffness during Bayesian search. I now find the paper quite convincing.
The authors would like to thank again the reviewer for the valuable comments and feedback that helped us to improve the clarity of the paper. The authors are happy to read that the previous answers reached the expectation of the reviewer.

3 Response to the Reviewer 4

The authors present a new method for 3D localization of nodules in sot tissues based on a variable stiffness probe equipped with F/T sensors for kinesthetic perception and a tactile array for tactile perception. The authors proposed an exploration strategy based on a Bayesian approach to the detection and localization of the nodule that allows the authors to set the stiffness of the probe and the direction of the sweep to detect and localize the nodule. The paper is well written and clear. However, I have some doubts and concerns that I would like the authors to clarify.

1. The proposed study is based on the experimental results over a simplified setup in which the soft tissue is isotropic and homogenous, and its surface is flat. Since the method relies on fine-tuning of a few thresholds, how does it generalize to the real case, where the conditions on the soft tissue are not so clean, and the presence of other organs and tissues (even as stiff as bones) affects the sensed force?

   The aim of this paper is to quantify the role of compliance in the probe to accurately estimate the depth of a stiff formation. Therefore, we idealized the scenario as much as possible to remove the effect of any other artefacts introduced by the tissue. We even use a spherical hard nodule to remove the effect of uneven geometry of the nodule. We do not claim that there is a specific probe stiffness that gives the best result irrespective of the probe and the tissue condition. Therefore, what can be generalized is that probe stiffness matters in conditioning the shape of the likelihood function (sensor model) in a Bayesian

framework to estimate a given feature in the tissue (in this case the nodule depth after having identified the location using lateral sweeps). Therefore, when the tissue is inhomogeneous, multiple force prominence values will be present in the force profile. Then a shape filter such as a convolution of a target shape over the force profile should be developed to extract the target feature. This is beyond the scope of this paper. We added the following text in the discussion to address this concern:

In section *Discussion*, paragraph 5 "*More generally, this paper concludes that the probe stiffness matters in conditioning the shape of the likelihood function (sensor model) in a Bayesian framework to estimate a given feature in the tissue (in this case the nodule depth after having identified the location using lateral sweeps). However, when the tissue is inhomogeneous, multiple force prominence values will be present in the force profile. Then a suitable technique should be adopted to filter a target force prominence shape. One realtime solution is to convolve a target force shape on the measured force data. A data-driven approach can be used to build the target shape from a variety of tissue samples with a given feature in them.*"

2. I don't see a great added value in the FEM simulations that the authors propose. First, I would like to have more details about how contacts were modeled (are there bearings, bushes, ... ?), and about the size of the soft tissue and its constraints to a fixed frame Second, if it can have a role in setting the initial value of the stiffness for a real case experiment, the soft tissue should be modeled more accurately.

   The FEM simulations were done to understand how the probe stiffness variation leads to changes in tissue stress dynamics that cannot be measured otherwise. Such insights are useful to understand why probe stiffness variation and resulting differences in the shape of likelihood functions are underpinned by the coupled dynamics between the probe and the tissue. Also, we have shown that the FE Analysis can be used to generate the likelihood function. Using this likelihood functions, generated from simulations, comes at a cost on the confidence level of the prediction, but can be useful in the case where no experimental palpation data is available. Regarding the contact models; unfortunately, COMSOL documentation does not specify if bearings or bushes models are used. However, the model is defined with 2 contact pairs: the fingertip and the phantom. Then the pressure contact calculation between the 2 contact pairs is based on an Augmented Lagrangian Method, and the friction between the 2 pairs is modeled as Coulomb friction. Finally, no rolling resistance is modeled for the contact between the probe and the phantom, which is equivalent to assuming pure sliding. The authors agree that there is still room for improvement in the soft tissue model. Yet, for the proposed application, the FE model is accurate enough to perform the 3D localization of the nodules based on the likelihood functions obtained from the simulation results. To improve the clarity of the model approach and support the interest of the proposed FE simulation, the following modifications have been added to the paper:

   In section *Finite Element Simulation*, paragraph 5 "*The contact between the probe and the phantom is modeled with two surface contact pairs covering the palmar region of the probe and the upper layer of the phantom, respectively. The pressure contact calculation is based on an Augmented Lagrangian Method, and the friction between the 2 contacts is modeled as Coulomb friction (μ in Table 1). Finally, no rolling resistance is modeled for the contact between the probe and the phantom assuming pure sliding at the elements level.*"

   In section *Discussion*, paragraph 2 "*Also, increasing the accuracy of the tissue model or simulating the lateral sweeps would require developing a 3D FE model and repeating the simulation for several shifts (position along* $\mathbf{x}$*). These modifications would increase the computational cost of the simulation significantly.*"

3. My third concern is about parameters. In particular the soft tissue thickness, the nodule Young modulus, and its depth. Since nodule diameter is 16mm, I would expect to have at least 32mm of soft tissue between the nodule and the supporting rigid plane. Even if the applied forces are small, it is a bit surprising that the nodule, especially at 2mm depth, does not influence the stress tensor of the surrounding soft tissue. For what regarding the depth, why the maximum selected depth is 8mm?

   The phantom has been designed with a similar ratio tissue thickness to nodule diameter than the ones used in published related works such as [25, 47, 29]. Also, it can be noticed that in the case of T1 tumors in the kidney or in the left lobe of the liver, for instance, the ratio between the organ thickness and tumor

thickness can be inferior to 2. As the reviewer noticed, the FE simulation shows that with the small forces applied during the longitudinal sweeps, the stress tensor under the nodule is not influenced even for the 2mm deep nodule. This phenomenon comes from the fact that during the palpation sweeps, the displacement of the nodule is negligible compared to the displacement of tissue above the nodule. In other words, the amount of tissue compressed between the probe and the nodule (referred in this article as the nodule depth) is more significant than the amount of tissue under the nodule. Finally, we decided to limit the nodule depth to 8mm to reduce the average palpation force level to minimise damage to the probe. Indeed, as shown in related works, detecting deeper nodules requires deeper indentation and by consequence, higher forces. Higher forces also lead to faster degradation of the tissue in repeated trials, making it difficult to compare results. Moreover, the lateral sweeps used an array of capacitive tactile sensors, that saturate if an excessive force is applied during lateral sweeps to locate the nodule. Since the development of a tactile sensor array for deep tissue exploration is not the focus of this paper, we used a nodule depth that meets all hardware requirements to demonstrate the key scientific phenomenon mentioned above. The discussion generated by the reviewer's comment have been added in the paper as follows:

In section *Soft tissue phantom with nodules*, paragraph 1 "*The ratio between the tissue thickness and the nodule diameter has been chosen accordingly to the one used in related studies in the literature [25, 47, 29]*"

In section *Soft tissue phantom with nodules*, paragraph 2 "*In the presented study, we limited the nodule depth to 8mm to reduce the average palpation force level and minimize damage to the probe. Indeed, as shown in related works, detecting deeper nodules requires deeper indentation and by consequence, higher forces. Higher forces also lead to faster degradation of the tissue in repeated trials, making it difficult to compare results. Moreover, the lateral sweeps used an array of capacitive tactile sensors, that saturates if an excessive force is applied to locate the nodule. To avoid saturating the tactile sensor, we used a nodule depth that meets all hardware requirements to demonstrate the role of stiffness variation in conditioning the haptic perception during 3D localization of nodules in soft tissues.* "

In section *Simulation results*, paragraph 2 "*Moreover, with the small forces applied during the longitudinal sweeps, the stress in the material under the nodule is not impacted as much as the stress in the material above the nodule. This phenomenon comes from the fact that during the palpation sweeps, the displacement of the nodule is small compared to the displacement of tissue above the nodule. This shows that the probe is more significantly affected by the amount of material above the nodule (the nodule depth) than the amount of material under the nodule.*"

4. The proposed algorithm seems quite sensitive to the thresholds. In all cases one lateral sweep allows the system to make the right decision whether the nodule is present or not. Did the authors try starting from a position far from the nodule? Is it so unlikely to have $P(N) < 0.2$ after the first sweep if it takes place far from the nodule?

   As any control strategy, the proposed algorithm relies on the tuning of some parameters (the threshold here). In the sake of clarity, the authors described and discuss the role of each chosen threshold. One of the advantages of the proposed threshold is that it is simple for the user to tune them according to their desired level of confidence. The authors proposed this threshold because it is the one that gave the best detection rate during the validation of the algorithm. The authors tested the algorithm at different starting distance from the nodule, and the detection was successful independently from the starting point. The limitation of this threshold comes more from the risk of false-positive than from missing a nodule due to the initial position of the probe. Indeed, the algorithm continues to investigate even if the probability of having a nodule is higher or equal 20%, which may result in some case in additional lateral exploration when it was not required. The discussion from this question has been added to the paper:

   In section *Evaluation of the algorithm*, paragraph 4 "*With the proposed threshold values, the algorithm was 100% accurate on the estimation of the presence of a nodule with a single lateral sweep. When a nodule was present, $P(N)$ was over 0.98 after the first sweep, independently from the initial distance between the probe and the nodule. When there was no nodule, P(N) was in a range between 0 and 0.18 after the first lateral sweep. A trade-off needs to be found while tuning P_N^{th} in order to maximize the*

detection rate but not increasing the number of false-positive detection, which would lead to unnecessary additional lateral explorations."

5. The authors claim submillimeter accuracy, but this is not supported by the results. Please note that it is even difficult to place the nodule in the soft body with such accuracy. Moreover, I can't see in the results where such accuracy has been achieved.

   The authors disagree on this point with the reviewer. Indeed, the Root Mean Square Error (RMSE) for the eight nodule detections using the presented algorithm (the two trials with fixed stiffness are removed since they do not represent the algorithm) is 0.27mm. The highest absolute error is 0.53mm for the 6mm deep nodule with the likelihood function obtained from the FE simulation. Both the RMSE and the maximum absolute error are under 1mm, which support the authors claim on submillimeter accuracy. The accuracy of the nodule detection is computed from the estimated depth computed from the probability distribution obtained after the last sweep (when the algorithm ends) as follow:

$$d_{est} = \sum_{d \in \mathscr{D}} P(d)d$$

   Of course, the computed errors relies on the fact that the position of the nodule tested to generate the likelihood functions are the ground truth for 2, 4, 6 and 8 mm deep. To better support the author claim, the following modifications have been added to the paper:

   In section *Evaluation of the algorithm*, paragraph 7 "*The final depth estimate d_{est} can be computed as follows:*

$$d_{est} = \sum_{d \in \mathscr{D}} P(d)d$$

   The Root Mean Square Error (RMSE) between the estimated depths and the actual nodule depths for all the detections presented in Fig 14 and Fig 15A is 0.27mm. The highest absolute error is 0.53mm for the 6mm deep nodule with the likelihood function obtained from the FE simulation."

6. Algorithm1: I suggest the inclusion of an escape from the do-while (e.g. a timeout). Line 15: shouldn't dir be "lat" instead of "long"? Line 18: shouldn't dir be "long" instead of "lat"?

   The authors would like to thanks the authors for his suggestion. Adding a timeout to the algorithm would probably increase the robustness of the algorithm for real-world scenarios. However, for the purpose of this paper, the author does not feel the need to add one since the threshold on the information gain already act like a timeout. Indeed when the information gain during a new sweep is too low, the algorithm stops the sweeps and returns the estimated position and depth probability distribution. Thank you very much for pointing out the typos for line 15 and 18, the directions have corrected.

7. line 221: what does it mean "implemented in real-time"?

   The authors were using inappropriately "real-time", thank you for pointing it out. The sentence has now been rephrased as follows:

   In section *Variable stiffness palpation: the VLM probe*, paragraph 4 "*The programs to run the experiment and the algorithm have been implemented using C++.*"

8. Typos: line 334: remove "performed", line 462: "sweeps", line 488: "cases", line 512: remove "distance"

   The authors would like to thank the reviewer once again for pointing these typos out. They have been corrected.
